# Data from the Baby Siblings Research Consortium confirm and specify the nature of the female protective effect in autism: A commentary on Messinger et al.

**DOI:** 10.1186/s13229-016-0092-x

**Published:** 2016-06-29

**Authors:** John N. Constantino

**Affiliations:** Blanche F. Ittleson Professor of Psychiatry and Pediatrics, William Greenleaf Eliot Division of Child Psychiatry, Washington University School of Medicine, 660 S. Euclid Avenue, Campus Box 8504, St. Louis, MO 63110 USA

**Keywords:** Female protective effect, Sex, Resilience, Compensatory, Autism spectrum disorder

## Abstract

Sibling recurrence data from the Baby Siblings Research Consortium (BSRC) recapitulate results from very large clinical family studies that demonstrate the absence of the Carter effect and provide clarification of the nature of the female protective effect in ASD. This legacy prospective data collection confirmed marked differences in the proportions of males versus females who lie along deviant trajectories of social development in the setting of inherited liability to autism—a phenomenon which defines the female protective effect—and demonstrate that among affected children, sex differences are modest and homologous to those observed among non-ASD children.

## Background

I read with great interest the report and analysis of the extensive collection of data by the Baby Siblings Research Consortium regarding early sex differences [1]. Given the rarity of such a large, prospective data collection (1241 infant siblings from 15 sites), these results will and should command considerable attention in the scientific community. It was, however, potentially misleading for the authors to conclude that the data did not support a “female protective effect” hypothesis, and this presents an opportunity to clarify the implications of the results as well as what is meant by the term “female protective effect.”

What the data suggested, replicating what has actually been previously reported by numerous prior studies [2, 3], is the absence of the so-called “Carter effect.” The Carter effect is the observation of the expectation that if a higher quantitative burden of genetic susceptibility is required to cross the threshold of affectation in one sex versus another, then one should observe a higher level of familial aggregation of the disorder among the relatives of probands of the sex that has the higher threshold. As is true for large registries in the USA and Europe [4–6], the Carter effect was *not* observed for ASD in the Baby Siblings Research Consortium (BSRC) sample. But the absence of the Carter effect is not the same as the absence of a female protective effect.

### Main Text

The manuscript describes the BSRC observation that across a number of developmental competencies, modest sex differences were observed, and the discrepancy between boys and girls with ASD was not different in magnitude from that between typically developing boys and girls. The authors conclude that therefore sex differences were “not autism-specific.” Examining males and females within the category of the disease-of-interest is an important exercise in understanding sex differences, but there can be profound ASD-specific sex effects on a disease that render sex differences among affected subjects relatively minor and indistinguishable from those differentiating typical boys and girls. Conduct disorder provides an excellent example—the differences between males and females who meet diagnostic criteria are minimal (by definition) and yet males are six times more likely to be affected than females. 

**Fig. 1:**
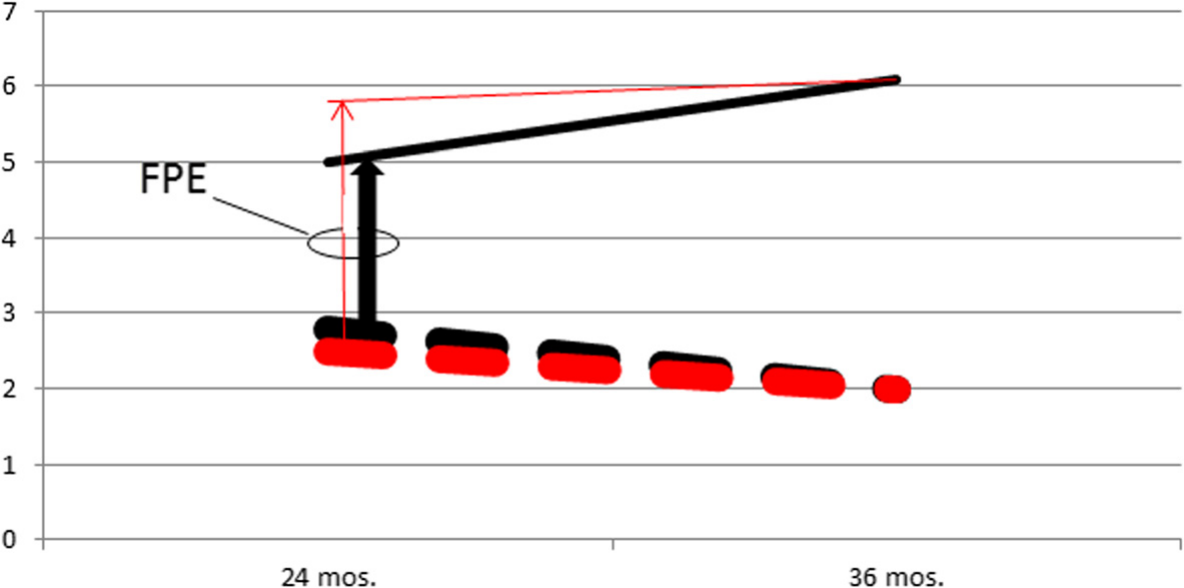
Schematic reconstruction of Figure 3 (panel A) of the original manuscript (Messinger et al. 2015). ADOS social affect scores of high-risk infant siblings (*n* = 1241, 58 % male), males in *black*, females in *red*; for children deemed “non-ASD”, *dashed lines*; children deemed “ASD”, *solid lines*. The panel is schematically redrawn to scale, with line *widths* depicting respective sample proportions. Whereas 193 males deviated enough from normality (*black arrow*) to contribute to the categorically diagnosed group, only 59 females deviated enough from normality (*red arrow*) to contribute to the categorically diagnosed group. The female protective effect is best represented by this proportional contrast (represented by the relative width of the arrows depicting the proportion for each sex who crossed over from typicality to abnormality) rather than by sex differences within each group

In the case of ASD, the first major clue generated by these data was that the magnitude of sex difference for any given trait *within* any subject group was *dwarfed* by the magnitude of difference *across* groups (ASD versus non-ASD). Since the “quantum leap” from typicality to clinical abnormality (the magnitude of which, in relation to within-group differences, is illustrated in figure) occurred over three times more commonly for male siblings than for female siblings (this was the second major clue generated by the study data), the data actually represent a remarkable manifestation of *sex-specific modulation of inherited liability*, which is the fundamental nature of the so-called female protective effect. The refocusing of the problem on contrasts *across* groups rather than *within* groups is represented by comparing the arrows and relative sample sizes in Fig. 1, which is a schematic reconstruction-to-scale of Figure three of the original manuscript.

In prior published work, we have presented evidence for the manner in which a female protective effect might operate in the absence of the Carter effect, essentially via a categorical mechanism of protection by which most females are protected from most forms of ASD [4, 7–9]. The BSRC data reported in the manuscript is entirely consistent with such modulation of risk by sex, and we have shown in quantitative trait analyses among school-aged children in both the AGRE and IAN registries how such protection manifests itself among females in multiplex autism families [4, 10].

## Conclusions

The BSRC data recapitulate results from very large clinical family studies that demonstrate the absence of the Carter effect and provide clarification of the nature of the female protective effect in ASD. The prospective data collection confirmed marked differences in the proportions of males versus females who lie along deviant trajectories of social development in the setting of inherited (autosomal) liability to autism—a phenomenon which *defines* the female protective effect—and demonstrate that *among* affected children, sex differences are modest and homologous to those observed among non-ASD children.

## References

[1] Messinger DS, Young GS, Webb SJ, Ozonoff S, Bryson SE, Carter A (2015). Early sex differences are not autism-specific: a Baby Siblings Research Consortium (BSRC) study. Mol Autism.

[2] Goin-Kochel RP, Abbacchi A, Constantino JN, Autism Genetic Resource Exchange Consortium (2007). Lack of evidence for increased genetic loading for autism among families of affected females: a replication from family history data in two large samples. Autism.

[3] Constantino JN (2014). Recurrence rates in autism spectrum disorders. JAMA.

[4] Constantino JN, Zhang Y, Frazier T, Abbacchi AM, Law P (2010). Sibling recurrence and the genetic epidemiology of autism. Am J Psychiatry.

[5] Grønborg TK, Schendel DE, Parner ET (2013). Recurrence of autism spectrum disorders in full- and half-siblings and trends over time: a population-based cohort study. JAMA Pediatr.

[6] Sandin S, Lichtenstein P, Kuja-Halkola R, Larsson H, Hultman CM, Reichenberg A (2014). The familial risk of autism. JAMA.

[7] Constantino JN, Todorov A, Hilton C, Law P, Zhang Y, Molloy E (2013). Autism recurrence in half siblings: strong support for genetic mechanisms of transmission in ASD. Mol Psychiatry.

[8] Constantino JN, Charman T (2012). Gender bias, female resilience, and the sex ratio in autism. J Am Acad Child Adolesc Psychiatry.

[9] Gockley J, Willsey AJ, Dong S, Dougherty JD, Constantino JN, Sanders SJ (2015). The female protective effect in autism spectrum disorder is not mediated by a single genetic locus. Mol Autism.

[10] Virkud YV, Todd RD, Abbacchi AM, Zhang Y, Constantino JN (2009). Familial aggregation of quantitative autistic traits in multiplex versus simplex autism. Am J Med Genet B Neuropsychiatr Genet.

